# Prognostic value of the PDW/HDL-C ratio in patients with chest pain symptoms and coronary artery calcification

**DOI:** 10.3389/fcvm.2022.824955

**Published:** 2022-07-25

**Authors:** Ya-Jing Qiu, Jun-Yi Luo, Fan Luo, Xin-Xin Tian, Lu Zeng, Zhuo-Ran Zhang, Xiao-Mei Li, Yi-Ning Yang

**Affiliations:** ^1^Department of Cardiology, First Affiliated Hospital of Xinjiang Medical University, Ürümqi, China; ^2^Xinjiang Key Laboratory of Cardiovascular Disease Research, Clinical Medical Research Institute of First Affiliated Hospital of Xinjiang Medical University, Ürümqi, China; ^3^Department of Cardiology, People’s Hospital of Xinjiang Uygur Autonomous Region, Ürümqi, China; ^4^People’s Hospital of Xinjiang Uygur Autonomous Region, Ürümqi, China

**Keywords:** chest pain, coronary artery calcification, PDW/HDL-C ratio, coronary heart disease (CHD), major adverse cardiovascular and cerebrovascular events (MACCEs)

## Abstract

**Background:**

Platelet-related parameters and HDL-C have been regarded as reliable and alternative markers of coronary heart disease (CHD) and the independent predictors of cardiovascular outcomes. PDW is a simple platelet index, which increases during platelet activation. Whether the PDW/HDL-C ratio predicts major adverse cardiovascular and cerebrovascular events (MACCEs) in patients who complained of chest pain and confirmed coronary artery calcification remains to be investigated. This study aimed to investigate the prognostic value of the PDW/HDL-C ratio in patients with chest pain symptoms and coronary artery calcification.

**Methods:**

A total of 5,647 patients with chest pain who underwent coronary computer tomography angiography (CTA) were enrolled in this study. Patients were divided into two groups according to their PDW/HDL-C ratio or whether the MACCE occurs. The primary outcomes were new-onset MACCEs, defined as the composite of all-cause death, non-fatal MI, non-fatal stroke, revascularization, malignant arrhythmia, and severe heart failure.

**Results:**

All patients had varying degrees of coronary calcification, with a mean CACS of 97.60 (22.60, 942.75), and the level of CACS in the MACCEs group was significantly higher than that in non-MACCE (P<0.001). During the 89-month follow-up, 304 (5.38%) MACCEs were recorded. The incidence of MACCEs was significantly higher in patients with the PDW/HDL-C ratio > 13.33. The K–M survival curves showed that patients in the high PDW/HDL-C ratio group had significantly lower survival rates than patients in the low PDW/HDL-C ratio group (log-rank test: *P* < 0.001). Multivariate Cox hazard regression analysis reveals that the PDW/HDL ratio was an independent predictor of MACCEs (HR: 1.604, 95% CI: 1.263–2.035; *P* < 0.001). Cox regression analysis showed that participants with a lower PDW/HDL-C ratio had a higher risk of MACCEs than those in the higher ratio group. The incidence of MACCEs was also more common in the PDW/HDL-C ratio > 13.33 group among different severities of coronary artery calcification. Furthermore, adding the PDW/HDL-C ratio to the traditional prognostic model for MACCEs improved C-statistic (*P* < 0.001), the NRI value (11.3% improvement, 95% CI: 0.018–0.196, *P* = 0.01), and the IDI value (0.7% improvement, 95% CI: 0.003–0.010, *P* < 0.001).

**Conclusion:**

The higher PDW/HDL-C ratio was independently associated with the increasing risk of MACCEs in patients with chest pain symptoms and coronary artery calcification. In patients with moderate calcification, mild coronary artery stenosis, and CAD verified by CTA, the incidence of MACCEs increased significantly in the PDW/HDL-C ratio > 13.33 group. Adding the PDW/HDL-C ratio to the traditional model provided had an incremental prognostic value for MACCEs.

## Introduction

The growing global burden of coronary heart disease (CHD) has long been at the forefront of the global health agenda (1, 2). Chest pain and other symptoms suggestive of CHD are common outpatient and emergency department symptoms in contemporary medical practice. Determining whether chest pain is due to CHD is imperative for directing appropriate symptomatic and preventive therapies. Guidelines have increasingly advocated the use of coronary computer tomography angiography (CTA) for the investigation of chest pain, particularly in those who have possible angina and no previous CHD, due to its ability to identify coronary artery calcification (3). Coronary calcification is an independent risk factor for future adverse events, reflecting coronary atherosclerotic burden, with this plaque burden in turn positively associated with progression (4).

Atherosclerosis is considered the predominant pathological basis of CHD, which is characterized by the development of lipid-rich plaques, in the artery wall. Platelets contribute to the early steps of plaque formation and participate in thrombus formation on ruptured plaque obviously (5), so platelet-related parameters are attractive indicators in the evaluation and prediction of CHD. Platelet distribution width (PDW) measures the variability in platelet size and is a more specific marker of platelet activation since it does not increase during simple platelet swelling (6). Previous studies have shown that HDL-C has a strong influence on the regulation of platelet production, metabolism, and activity, and has antiplatelet action, which affects the occurrence of cardiovascular adverse events (7–9).

Exploring new reference indicators appears to have a significant value not only for atherosclerosis itself, but also for adverse high-risk plaque characteristics and prognosis of CHD. Whether the combined index of platelet and HDL-C can be a new reference indicator needs further research and analysis. To address the knowledge gap, this study aimed to specifically investigate whether the PDW/HDL ratio has a prognostic value for major adverse cardiovascular and cerebrovascular events (MACCE) in patients with chest pain and coronary artery calcification.

## Materials and methods

### Study population

**FIGURE 1:**
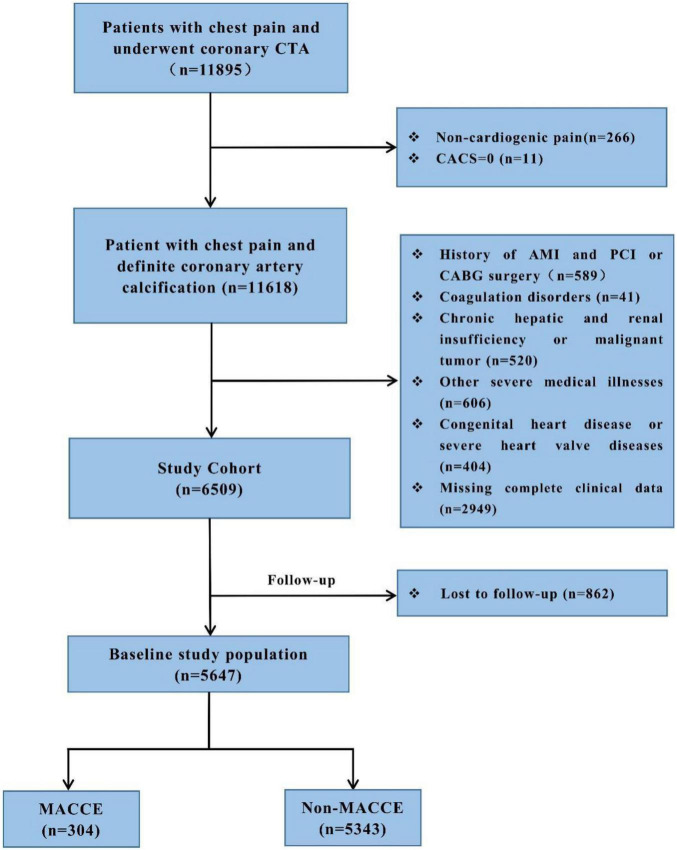
Flow diagram of this retrospective.

We performed a retrospective cohort study in the First Affiliated Hospital of Xinjiang Medical University, from January 2014 to October 2020. This study was approved by the local research ethics committee. Given the retrospective nature of this research, no informed consent was required. All these patients complained of chest pain and underwent CTA detection which diagnosed them with coronary artery calcification (CACS > 0). The study excluded people who had non-cardiac chest pain (*n* = 277) and myocardial infarction and underwent PCI or CABG surgery (*n* = 589), coagulation disorders (*n* = 41), chronic hepatic and renal insufficiency or malignant tumor (*n* = 520), congenital heart disease or severe heart valve diseases (*n* = 404), other severe medical illnesses (*n* = 606), and insufficient clinical data (*n* = 2,949). A total of 6,509 patients were followed up from January 2014 to April 2021 by telephone or outpatient clinical visit each year, and 5,647 (86.8%) patients completed the follow-up. Finally, a total of 5,647 participants were included in the study. The detailed recruitment process is depicted in Figure 1. All patients were given antiplatelet and statin therapy for the secondary prevention of CHD. Outpatient or telephone follow-up was performed at 1, 3, 6, and 12 months after discharge, and once a year thereafter.

### Data collection and risk factor definitions

Clinical data were collected from all of the medical records by trained clinicians who were blinded to the purpose of the study. The data included age, gender, smoking history, history of T2DM and hypertension, family history of CHD, blood pressure (SBP and DBP), heart rate (HR), and coronary artery calcification score (CACS), and medication at discharge. Peripheral venous blood samples were collected early in the morning after an overnight fast on admission and analyzed shortly after sampling. Total cholesterol (TC), triglycerides (TG), high-density lipoprotein cholesterol (HDL-C), low-density lipoprotein cholesterol (LDL-C), fasting blood glucose (FBG), platelet count (PC), mean platelet volume (MPV), PDW, and platelet crit (PCT) levels were analyzed. All the patients underwent the first CTA during this hospitalization.

Type 2 diabetes mellitus (T2DM) was defined as a history of diabetes or intake of antidiabetic medication. Hypertension was defined as the current use of anti-hypertensive medications or a previous diagnosis. Smoking was defined as either active smoking or having a previous smoking history. Family history of CHD was defined as having more than 3 family members in the immediate or collateral family with a history of CHD.

### Angiography and severity of coronary heart disease

Those CTA were uniformly acquired by using multi-detector row CT scanners consisting of 64-rows or greater. CACS was measured by using the scoring system developed by Agatston. For this investigation, we evaluated the severity of coronary artery calcification from three different perspectives as follows: severity of coronary calcification (CACS ≤ 100, CACS 100-400, and CACS > 400), extent of coronary stenosis, and obstructive disease classification. The extent of coronary stenosis was categorized as “mild stenosis” (<50% luminal stenosis), “moderate stenosis” (50 – 75% luminal stenosis), and “severe stenosis” (>75% luminal stenosis). The obstructive disease was categorized as “no CAD” (absence of any plaque), “non-obstructive CAD” (all coronary arteries < 50% luminal stenosis), or “obstructive CAD” (at least one artery > 50% luminal stenosis).

### Endpoints

The primary endpoint was new-onset MACCEs, defined as the composite of all-cause death, non-fatal MI, non-fatal stroke, revascularization, malignant arrhythmia, and severe heart failure. All-cause death referred to the death attributed to cardiovascular or non-cardiovascular causes.

### Statistical analysis

Data analysis was carried out using SPSS 19.0 and R version 4.1.1. Continuous variables were reported as mean ± standard deviation when normally distributed. The CACS, TG, FBG, and PDW were not normally distributed; therefore, those variables were expressed as medians with interquartile ranges. Categorical variables were expressed as frequencies. Two-samples independent *t*-test, Mann–Whitney test, and chi-square test were used to investigate for any statistically significant difference between the baseline characteristics. Survival analysis was performed by using the Kaplan Meier’s survival curves in the followed up patients from the time of enrollment until the time of their death for the deceased, and until the time of follow-up for the survivors. Univariate and multivariate stepwise Cox proportional hazard regression analyses were constructed to identify the independent predictors of MACCEs. Cox regression analysis was also used for predicting MACCEs after adjusting conventional cardiovascular risk factors such as age and T2DM, and we stratified the analyses by (1) severity of coronary artery calcification, (2) extent of coronary stenosis, and (3) obstructive disease classification. Multivariate analysis of variance was used to compare the incidence of MACCEs in the high and low ratio groups with different degrees of coronary artery calcification. Finally, the ability of the PDW/HDL-C ratio to improve MACCE prediction beyond using traditional risk factors was assessed by using the indexes of discrimination as measured by the c-statistic and integrated discrimination improvement (IDI), and reclassification as measured by the category-less net reclassification index (NRI).

## Results

### Baseline characteristics of patients

The study patients had an average age of 62.55 ± 10.39 years and 3,740 (66.2%) patients were male. During the 89-month follow-up, 304 (5.38%) MACCEs were recorded. According to this, we stratified our population by whether MACCEs occur and found that the age was markedly higher in the MACCEs group. Baseline clinical characteristics and clinical event data of the two groups are listed in Table 1. Patients with MACCEs had higher levels of the CACS, TG, FBG, PDW, and PDW/HDL-C ratio, and lower HDL-C levels than the non-MACCEs group. There were no significant differences in drug therapies between the two groups (*P* < 0.05).

**TABLE 1:** Comparison of baseline characteristics between MACCE and Non-MACCE groups.

Variable	Overall cohort	Non-MACCE	MACCE	*P*
	*n* = 5647	*n* = 5343	*n* = 304	
Age (years)	62.55 ± 10.39	62.35 ± 10.34	66.09 ± 10.64	<0.001
Male	3740 (66.2%)	3526 (65.9%)	214 (70.3%)	0.114
Smoker	1931 (34.2%)	1828 (34.2%)	103 (33.9%)	0.906
T2DM	1868 (33.1%)	1751 (32.8%)	117 (38.5%)	0.039
Hypertension	3945 (69.9%)	3727 (69.8%)	218 (71.7%)	0.470
Family history of CAD	816 (14.4%)	776 (14.5%)	38 (12.5%)	0.328
SBP (mmHg)	130 ± 18	130 ± 18	130 ± 19	0.833
DBP (mmHg)	78 ± 11	78 ± 11	77 ± 11	0.186
HR (bpm)	77 ± 10	77 ± 10	77 ± 11	0.270
CACS	97.60 (22.60, 942.75)	93.20 (21.50, 329.30)	234.15 (57.50, 787.05)	<0.001
TC (mmol/L)	4.18 ± 1.11	4.18 ± 1.10	4.14 ± 1.22	0.473
TG (mmol/L)	1.59 (1.12, 2.39)	1.60 (1.12, 2.40)	1.48 (1.03, 2.24)	0.017
HDL-C (mmol/L)	1.14 ± 0.33	1.14 ± 0.33	1.07 ± 0.29	<0.001
LDL-C (mmol/L)	2.71 ± 0.94	2.71 ± 0.93	2.69 ± 1.01	0.718
FBG (mmol/L)	5.43 (4.76, 6.89)	5.41 (4.75, 6.87)	5.61 (4.88, 7.35)	0.012
PC (10^9/L)	224.78 ± 61.33	224.83 ± 60.83	223.79 ± 69.63	0.798
PDW (%)	15.55 (12.30, 16.64)	15.55 (12.20, 16.60)	16.09 (13.30, 16.90)	<0.001
MPV (fL)	10.52 ± 1.17	10.52 ± 1.66	10.62 ± 1.22	0.138
PCT (%)	0.23 ± 0.06	0.23 ± 0.06	0.23 ± 0.07	0.999
PDW/HDL-C ratio	13.23 (10.34, 16.56)	13.16 (10.27, 16.42)	14.50 (11.71, 18.60)	<0.001
**Medication**				
Aspirin	1992 (35.3%)	1917 (35.9%)	75 (24.7%)	<0.001
β-blocker	1838 (32.5%)	1758 (32.9%)	80 (26.3%)	0.017
ACEI/ARB	1599 (28.3%)	1543 (28.9%)	56 (18.4%)	<0.001
Statin	3089 (54.7%)	2967 (55.5%)	122 (40.1%)	<0.001
CCB	1455 (25.8%)	1417 (26.5%)	38 (12.5%)	<0.001

The optimal PDW/HDL-C ratio cutoff value for predicting MACCEs was 13.33, which was calculated from sensitivity, specificity, and area under the curve (AUC) values determined by receiver operating characteristic analysis (AUC = 0.59, *P* < 0.001). Accordingly, participants were divided into the higher ratio group (PDW/HDL-C ratio > 13.33) and the lower ratio group (PDW/HDL-C ratio < 13.33). Baseline clinical characteristics and clinical event data of the two groups are listed in Table 2.

**TABLE 2:** Comparison of baseline characteristics between high and low ratio groups.

Variable	Overall cohort	PDW/HDL-C ratio < 13.33	PDW/HDL-C ratio ≥13.33	*P*
	*n* = 5647	*n* = 2866	*n* = 2781	
Age,years	62.55 ± 10.39	64 ± 10.21	61.01 ± 10.37	<0.001
Male	3740(66.2%)	1642(57.3%)	2098(75.4%)	<0.001
Smoker	1931(34.2%)	822(28.7%)	1109(39.9%0	<0.001
T2DM	1868(33.1%)	819(28.6%0	1049(37.7%0	<0.001
Hypertension	3945(69.9%)	1973(68.8%0	1972(70.9%0	0.090
Family history of CAD	816(14.4%)	400(14.0%)	414(14.9%)	0.320
SBP,mmHg	130 ± 18	129 ± 18	130 ± 18	0.204
DBP,mmHg	78 ± 11	77 ± 11	78 ± 12	<0.001
HR,bpm	77 ± 10	77 ± 10	77 ± 10	0.849
CACS	97.60(22.60,942.75)	98.35(21.50,328.50)	96.40(23.20,362.20)	0.366
LAD	51.60(11.90,117.95)	54.30(11.90,177.80)	48.80(12.00,181.40)	0.589
LCX	0.00(0.00,26.20)	0.00(0.00,24.10)	0.00(0.00,27.90)	0.015
RCA	3.40(0.00,61.70)	0.00(0.00,27.90)	4.90(0.00,72.80)	0.010
TC,mmol/L	4.18 ± 1.11	4.34 ± 1.11	4.02 ± 1.08	<0.001
TG,mmol/L	1.59(1.12,2.39)	1.44(1.02,2.11)	1.78(1.25,2.69)	<0.001
HDL-C,mmol/L	1.14 ± 0.33	1.34 ± 0.31	0.93 ± 0.19	<0.001
LDL-C,mmol/L	2.71 ± 0.94	2.84 ± 0.95	2.57 ± 0.90	<0.001
FBG,mmol/L	5.43(4.76,6.89)	5.36(4.74,6.66)	5.49(4.78,7.10)	<0.001
PC,10^9/L	224.78 ± 61.33	234.40 ± 60.19	214.86 ± 60.92	<0.001
PDW,%	15.55(12.30,16.64)	13.00(11.20,15.86)	16.28(15.30,17.10)	<0.001
MPV,fL	10.52 ± 1.17	10.28 ± 0.99	10.77 ± 1.28	<0.001
PCT,%	0.23 ± 0.06	0.24 ± 0.06	0.23 ± 0.06	<0.001
Medication				
Aspirin	1992(35.3%)	1035(36.1%0	957(34.4%)	0.181
β-blocker	1838(32.5%)	918(32.0%)	920(33.1%)	0.399
ACEI/ARB	1599(28.3%)	817(28.5%)	782(28.1%0	0.747
Statin	3089(54.7%0	1578(55.1%)	1511(54.3%)	0.584
CCB	1455(25.8%)	716(25.0%)	739(26.6%)	0.172
MACCEs	304(5.3%)	111(3.8%)	193(6.9%)	<0.001

### Higher PDW/HDL-C ratio indicates a higher major adverse cardiovascular and cerebrovascular event risk

**FIGURE 2:**
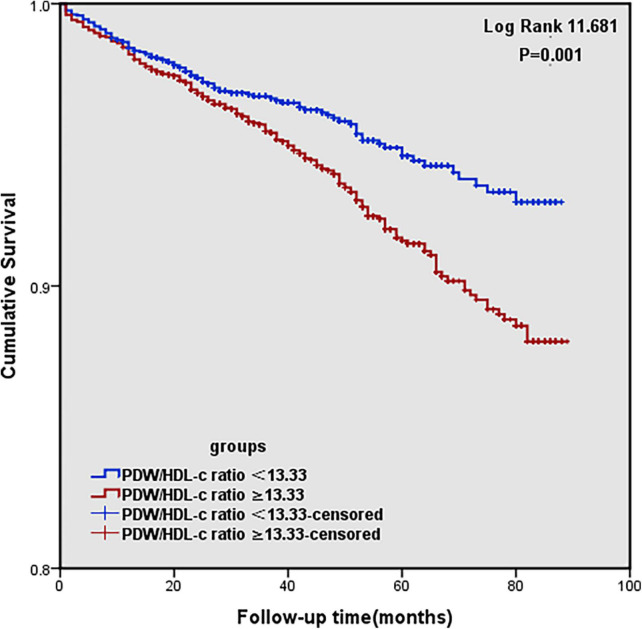
Kaplan–Meier survival curve for MACCEs (cardiovascular and cerebrovascular events) according to high and low PDW/HDL-C ratio groups.

The Kaplan–Meier survival analysis reveals that the higher the PDW/HDL-C ratio was, the lower the survival rate would be (log-rank:11.681, *P* = 0.001) (Figure 2).

Univariate and multivariate Cox proportional hazard regression analysis and predictors for MACCEs are presented in Table 3. In the univariate analysis, the criteria associated with MACCE occurrence were age, T2DM, CACS, FBG, PDW/HDL-C ratio, and treatment protocols. After adjusting confounding factors, including age, T2DM, and FBG, multivariate Cox proportional hazard regression analysis showed that the PDW/HDL-C ratio (HR: 1.604, 95% CI: 1.263–2.035, *P* < 0.001) and CACS (HR: 1.484, 95% CI: 1.293–1.703, *P* < 0.001) independently predicted the occurrence of MACCEs in patients.

**TABLE 3:** Univariate and multivariate Cox regression analysis for predicting MACCE.

Variables	Univariate HR (95% CI)	*P*	Univariate HR	Multivariate HR (95% CI)	*P*	Multivariate HR
Age (years)	1.035 (1.023,1.047)	<0.001	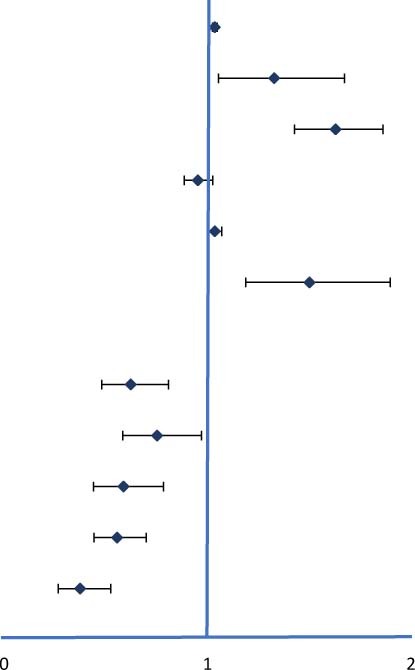	1.029 (1.017,1.041)	<0.001	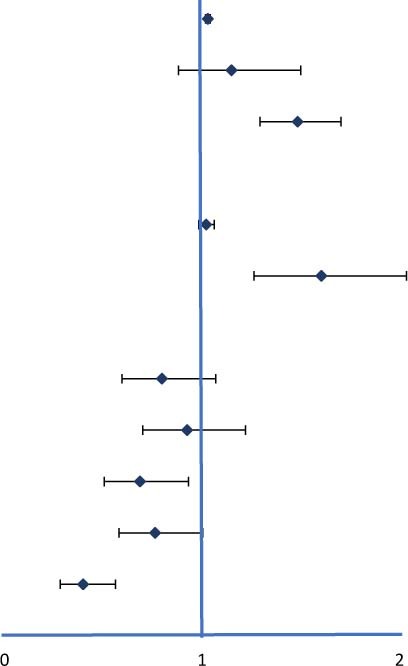
T2DM	1.327 (1.053,1.672)	0.016		1.149 (0.880,1.499)	0.307	
CACS	1.629 (1.427,1.861)	<0.001		1.484 (1.293,1.703)	<0.001	
TG (mmol/L)	0.952 (0.884,1.025)	0.193				
FBG (mmol/L)	1.035 (1.002,1.069)	0.035		1.021 (0.983,1.062)	0.282	
PDW/HDL-C ratio	1.500 (1.187,1.897)	0.004		1.604 (1.263,2.035)	<0.001	
medication						
aspirin	0.622 (0.479,0.808)	<0.001		0.797 (0.595,1.069)	0.129		
β-blocker	0.751 (0.582,0.970)	0.028		0.924 (0.700,1.219)	0.576	
ACEI/ARB	0.586 (0.438,0.783)	<0.001		0.686 (0.505,0.931)	0.016	
Statin	0.555 (0.441,0.698)	<0.001		0.762 (0.579,1.003)	0.052	
CCB	0.373 (0.266,0.524)	<0.001		0.398 (0.282,0.562)	<0.001	

Adjusted variables were age, T2DM, CACS, FBG, PDW/HDL-C ratio, aspirin, β-blocker, ACEI/ARB, statin and CCB.

Table 4 shows the 89-month event rate and Cox proportional hazard analysis for MACCEs in the two groups. The events include 140 (46.05%) all-cause death, 15 (4.93%) non-fatal MI, 31 (10.2%) non-fatal stroke, 109 (35.86%) revascularization, 5 (1.64%) malignant arrhythmia, and 4 (1.32%) severe heart failure. Upon unadjusted Cox modeling, the rate of revascularization and MACCEs rose significantly with the higher PDW/HDL-C ratio. After adjusting for age, T2DM, CACS, FBG, and drug therapies, a higher PDW/HDL-C ratio was the risk factor for the incidence of revascularization (HR:1.950, 95% CI: 1.299–2.928, *P* = 0.007) and MACCEs (HR:1.604, 95% CI: 1.263–2.035, *P* < 0.001).

**TABLE 4:** Baseline PDW/HDL ratio and prediction of MACCEs.

End point	Baseline PDW/HDL ratio	Events (n/total)	89 months event rate (%)	Unadjusted HR (95% CI)	*P*	Adjusted HR (95% CI)	*P*
All-cause death	low ratio	53/2866	1.85				
	high ratio	87/2781	3.13	1.386 (0.984–1.954)	0.062		
Non–fatal MI	low ratio	4/2866	0.14				
	high ratio	11/2781	0.4	2.373 (0.753–7.480)	0.140		
Non-fatal stroke	low ratio	16/2866	0.56				
	high ratio	15/2781	0.54	0.795 (0.392–1.614)	0.526		
Revascularization	low ratio	34/2866	1.19				
	high ratio	75/2781	2.7	1.950 (1.299–2.928)	0.001	1.774 (1.171–2.686)	0.007
Malignant Arrhythmia	low ratio	2/2866	0.07				
	high ratio	3/2781	0.11	1.363 (0.227–8.201)	0.735		
Severe heart failure	low ratio	2/2866	0.07				
	high ratio	2/2781	0.07	0.996 (0.140–7.073)	0.997		
MACCEs	low ratio	111/2866	3.87				
	high ratio	193/2781	6.94	1.500 (1.187–1.897)	0.001	1.604 (1.263–2.035)	<0.001

### The major adverse cardiovascular and cerebrovascular events were also more common in PDW/HDL-C ratio > 13.33 group among different severities of coronary artery calcification

**FIGURE 3:**
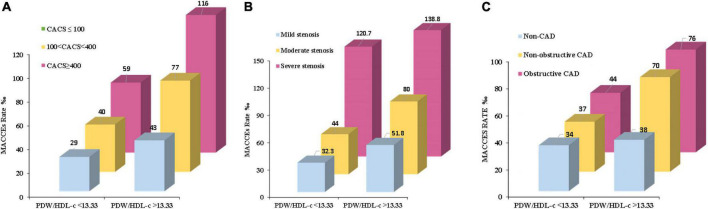
The MACCE rate in high and low PDW/HDL-C ratio groups among different severities of CHD. **(A)** Patients were stratified by CTA in the severity of coronary artery calcification (CACS ≤ 100, CACS 100-400 and CACS > 400). **(B)** Patients were stratified by CTA in the extent of coronary stenosis (mild stenosis < 50% luminal stenosis, moderate stenosis 50% – 75% luminal stenosis, and severe stenosis > 75% luminal stenosis). **(C)** Patients were stratified by CTA in the obstructive disease classification (no CAD: absence of any plaque, non-obstructive CAD: all coronary arteries < 50% luminal stenosis, and obstructive CAD: at least one artery > 50% luminal stenosis). Multivariate analysis of variance was used to compare the incidence of MACCEs in high and low ratio groups at different stratification.

Multivariate analysis of variance showed that the rate for MACCEs increased with the PDW/HDL-C ratio level and further increased with the severity of coronary artery calcification (P<0.001) (Figure 3A), especially in patients of CACS 100-400, yielding a higher event risk in those with the PDW/HDL-C ratio > 13.33 (HR: 1.039, 95% CI: 1.006–1.073, *P* = 0.019) (Table 5).

**TABLE 5:** PDW/HDL-C predicting MACCEs among different severity of coronary artery calcification.

	HR	95%CI	*P*
**The severity of coronary artery calcification**			
CACS ≤ 100	1.022	0.986–1.060	0.228
100<CACS ≤ 400	1.039	1.006–1.073	0.019
CACS>400	1.020	0.990–1.050	0.190
**The extent of coronary stenosis**			
Mild stenosis	1.032	1.003–1.063	0.033
Moderate stenosis	1.012	0.980–1.046	0.453
Severe stenosis	1.030	0.990–1.072	0.140
**Obstructive disease classification**			
No CAD	1.022	0.959–1.047	0.922
Non-obstructive CAD	1.043	1.006–1.082	0.022
Obstructive CAD	1.028	1.001–1.056	0.040

A similar phenomenon has been observed in patients stratified by the extent of coronary stenosis. As shown in Figure 3B, with the aggravation of vascular stenosis and the higher PDW/HDL-C ratio, the incidence of MACCEs increased remarkably (*P* < 0.001). It was especially obvious in the group of mild stenosis that the people with a high PDW/HDL-C ratio had a higher risk of developing MACCEs (HR: 1.032, 95% CI: 1.003–1.063, *P* = 0.033) (Table 5).

The equivalent result was obtained after patients were stratified by severity of coronary artery obstruction (*P* < 0.001) (Figure 3C). Compared to the lower ratio group, the MACCEs occurred more frequently in the higher ratio group both in patients with non-obstructive CAD (HR:1.043, 95% CI: 1.006–1.082, *P* = 0.022) and obstructive CAD (HR: 1.028,95% CI: 1.001–1.056, *P* = 0.040). Nevertheless, this result was not obvious in patients diagnosed with non-CAD by CTA (Table 5).

### The predictive ability was enhanced as the PDW/HDL-C ratio was added to the traditional model

The incremental predictive value of the PDW/HDL-C ratio for MACCEs is shown in Table 6. Adding the PDW/HDL-C ratio to the model of established risk factors improved the prediction of MACCEs (C-Statistic: 0.697, 95% CI: 0.665–0.729, *P* < 0.001). Moreover, the addition of the PDW/HDL-C ratio had an incremental prognostic value for predicting MACCEs in terms of NRI (11.3% improvement, 95% CI: 0.018–0.196, *P* = 0.01) and IDI (0.7% improvement, 95% CI: 0.003–0.010, *P* < 0.001), especially when compared with established risk factors and models adding single indicators.

**TABLE 6:** Evaluation of predictive models for MACCEs.

	C-Statistic	*P*	NRI (95% CI)	*P*	IDI (95% CI)	*P*
Established risk factors	0.683 (0.650–0.716)	<0.001	——	——	——	——
Established risk factors + PDW	0.69 (0.659–0.722)	<0.001	0.053 (−0.024–0.164)	0.304	0.003 (0.001–0.005)	0.002
Established risk factors + HDL-C	0.695 (0.663–0.727)	<0.001	0.109 (0.019–0.221)	0.030	0.006 (0.003–0.009)	<0.001
Established risk factors + PDW/HDL ratio	0.697 (0.665–0.729)	<0.001	0.113 (0.018–0.196)	0.010	0.007 (0.003–0.010)	<0.001

## Discussion

This study investigated the association between the PDW/HDL-C ratio and MACCEs in patients with chest pain symptoms and coronary artery calcification. The results showed that the higher PDW/HDL-C ratio was positively associated with increased MACCEs. The PDW/HDL-C ratio was an independent predictor of MACCEs after adjusting for confounding factors. Furthermore, our results showed that adding the PDW/HDL-C ratio to the predictive model may improve the discrimination of risk prediction for MACCEs in patients complaining of chest pain and who underwent CTA detection which diagnosed them with coronary artery calcification. These findings reveal the prognostic value of the PDW/HDL-C ratio for MACCEs in patients with chest pain and coronary artery calcification. To the best of our knowledge, this study demonstrated, for the first time, that the PDW/HDL-C ratio is a potential predictor for MACCEs. Most importantly, this study suggests that a simple, convenient, and cost-effective method may optimize the prediction of recurrent cardiovascular risk in patients with chest pain and coronary artery calcification.

Platelets play a pivotal role in coronary artery calcification, which is the main pathological change and primary cause of CHD. Variations and heterogeneity of blood cell shape are expressed by PDW for platelets (10). Lower levels of PDW were significantly related to lower risks of CHD (11). A study suggests that PDW may be associated with the degree of collateral development in chronic stable coronary artery disease (12). Another study confirmed that PDW can predict the presence of CTO (13). It is now well established from a variety of studies that HDL-C is negatively correlated with the incidence of CAD, and low concentrations of HDL-C indicate increased cardiovascular risk (14). In parallel, a higher HDL-C level was associated with better survival outcomes in CAD patients complicated by heart failure or diabetes (9, 15). It is now well established from a variety of studies that HDL-C can not only directly stimulate megakaryocyte progenitor cells resulting in platelet production (16), but also influence the function of SR-BI to maintain the normal platelet function and prevention of thrombosis (8, 17, 18). Due to the inextricable interaction between platelet and HDL-C in the pathogenesis of atherosclerosis, we contemplated that their combination indices would have great clinical significance for improving cardiovascular risk prediction, and should be the subject of intensive investigations both in humans and in animal models. A recent study observes that PLT/HDL-C ratio (PHR) is considered an independent variable for the prediction of the Gensini score in patients with an initial diagnosis of CHD (19). Another study demonstrates that PHR is significantly increased in patients with nascent metabolic syndrome (MetS) and appears to be a valid biomarker of MetS. It could also emerge as a biomarker for athero-thrombotic risk (20). However, there is no consensus on whether the PDW/HDL-C ratio could predict cardiovascular risks or not. Therefore, our study was the first to propose a correlation between the PDW/HDL-C ratio and MACCEs in patients with chest pain symptoms and coronary artery calcification. Considering the calculation design principle of the PDW/HDL-C ratio, the higher the PDW or the lower the HDL-C is, the higher the ratio will be. That is to say, a higher ratio means a higher risk of cardiovascular and cerebrovascular events. Thus, to the same extent, our study supported the above theories and previous related studies. Nevertheless, whether the PDW/HDL-C ratio is able to predict cardiovascular outcomes is currently lacking in patients with chest pain symptoms and coronary artery calcification.

To the best of our knowledge, the population in this study represents a relatively large cohort of patients with chest pain symptoms and coronary artery calcification in which the association between the PDW/HDL-C ratio and long-term MACCEs has been investigated. Moreover, our study is the first to report all-cause death, non–fatal MI, non-fatal stroke, revascularization, malignant arrhythmia, and severe heart failure as composite endpoint events. Additionally, patients in our study had higher percentages of non-fatal stroke and revascularization, thus denoting higher risk patients of cardiovascular and cerebrovascular diseases. Our study demonstrated that a higher PDW/HDL-C ratio was significantly associated with a higher risk of MACCEs. The higher risk of MACCEs persisted after adjusting for traditional cardiovascular risk factors, the burden of comorbidities, disease severity, and medications. We also found that the PDW/HDL-C ratio remained independently predictive of adverse cardiovascular and cerebrovascular outcomes after adjusting for important variables and treatment strategies. Although the PDW/HDL-C ratio independently predicted cardiovascular events in patients with chest pain and coronary artery calcification, the association between the PDW/HDL-C ratio and these individual events has not been well demonstrated. The possible reasons for these results are as follows: (1) after coronary artery calcification is diagnosed by CTA, the PDW/HDL-C ratio may not predict the risk of future myocardial infarction, non-fatal stroke, malignant arrhythmia, or severe heart failure in patients with suspected CHD. (2) In this study, the baseline PDW/HDL-C ratio was used to predict MACCEs, and changes in secondary preventive measures including oral drug therapy, diet, lifestyle, etc., in patients after discharge may affect the prediction of MACCEs by the PDW/HDL-C ratio. (3) During the study period, we experienced COVID-19, and some patients went to the local hospital when their disease progressed due to epidemic prevention and control. Therefore, some patients with MACCEs were not recorded by us. This may result in the relatively small number of events observed in our study subjects, so it is difficult to determine the exact relationship between the PDW/HDL-C ratio and these individual events.

Identifying and diagnosing coronary CHD in patients with chest pain is one of the great challenges faced by clinicians. Although coronary angiography is the gold standard for evaluating CHD, more interest is currently focused on the use of non-invasive imaging methods, such as CTA. CTA has been proved to be an effective tool for the rapid and accurate diagnosis of CHD in patients with low to moderate cardiovascular risk. In addition, CTA has a unique ability to non-invasively depict the anatomical structure of the coronary artery, allowing not only the visualization of the arterial lumen to detect severe stenosis or occlusion that causes myocardial ischemia but also to evaluate the coronary artery wall by showing the presence or absence of CAD (21–23). Recent research has shown that the first-line CTA facilitates more appropriate use of downstream use of statin and antiplatelet therapy than functional testing in patients with chest pain (24, 25). Given that CTA is increasingly used to evaluate symptomatic patients, our results have clinical implications for the prognostic analysis of patients who have chest pain with evidence of CAC and coexisting risk factors. In our study, we demonstrated that the severity of CAD is strongly associated with the PDW/HDL-C ratio and MACCEs during the follow-up. We estimated that the effect of the PDW/HDL-C ratio to predict outcomes was substantial in patients with CACS 100-400, mild stenosis, non-obstructive CAD, or obstructive CAD. Although the obstructive disease is not a direct marker of atherosclerosis burden, we chose this measure, as it was easy to obtain from CTA and it was highly correlated with the atherosclerotic burden (26, 27). Of note, the result was only applied to patients with moderate calcification, mild coronary artery stenosis, and CHD verified by CTA. This indicated that the prognostic value of the PDW/HDL-C ratio may not apply to the entire range of chest pain and coronary artery calcification. We speculate that this could be due to patients with massive severity of coronary calcification or severe stenosis receiving sustained medical treatment or surgery, which may attenuate the association between PDW/HDL-C ratio and MACCEs.

In this study, we identified the optimal PDW/HDL-C ratio cutoff value for predicting MACCEs. We found that the AUC of the optimal cutoff value of 13.33 was relatively poor, suggesting that it is difficult to predict hard endpoint events based on the PDW/HDL-C ratio alone. However, by adding the PDW/HDL-C ratio to the established risk factors of MACCEs, we found a significant improvement in risk prediction in terms of the C-statistic value, NRI, and IDI. Although previous studies have shown that a higher PDW level or a lower HDL-C level is associated with worse cardiovascular outcomes, none have discriminated against the incremental prognostic value of the PDW/HDL-C ratio in terms of hard clinical endpoints. Our results implied that the use of the PDW/HDL-C ratio may refine the risk stratification of cardiovascular risk. Routinely introducing the PDW/HDL-C ratio into clinical diagnostic models could more accurately identify patients with higher cardiovascular risk, thus enabling a more targeted treatment or prevention.

An increasing number of studies have shown that PDW and HDL-C play an indispensable role in the occurrence and development of CHD, and there is a close relationship between PDW and HDL (17, 18, 28). Hence, it is presumed that the factors mentioned above might be partially responsible for the observed relationship between the PDW/HDL-C ratio and MACCEs. More importantly, as mentioned in this study, the PDW/HDL-C ratio was positively associated with the severity of coronary artery calcification, suggesting that a difference in the extent of coronary atherosclerosis diagnosed by CTA may contribute to the graded PDW/HDL-C ratio-MACCE relationship, which will bring more convenience and practicability for clinical applications. Still, the exact mechanisms accounting for the association between the PDW/HDL-C ratio and MACCEs remain unclear, and these conclusions need to be further confirmed in prospective studies.

### Study limitations

This study has several potential limitations. First, platelet parameters and lipid levels were measured only at baseline. The PDW/HDL-C ratio may change with follow-up. Therefore, it is not clear whether the changes in PDW/HDL-C ratio predict cardiovascular outcomes. Second, data on other non-statin lipid-lowering drugs and antiplatelet aggregation drugs have not been recorded, which may have potential effects on the PDW/HDL-C ratio levels and cardiovascular outcomes. Third, all patients were patients with chest pain and coronary artery calcification verified by CTA, so the applicability of the conclusions of this study needs further study to examine those patients with no clinical symptoms and no coronary artery calcification. Finally, the subjects of the study were Chinese patients, so these results need to be repeated in other ethnic groups. Despite these limitations, this study has important clinical implications because it is the first to investigate the association between the PDW/HDL-C ratio and MACCEs in patients with chest pain symptoms and coronary artery calcification.

## Conclusion

The higher PDW/HDL-C ratio was independently associated with the increasing risk of MACCEs in patients with chest pain symptoms and coronary artery calcification. Identically, from different perspectives of the severity of coronary artery calcification, the same result was confirmed particularly in patients with moderate calcification, mild coronary artery stenosis, and CHD verified by CTA. Finally, adding the PDW/HDL-C ratio to the traditional model had an incremental prognostic value for MACCEs. These findings suggested that the PDW/HDL-C ratio is likely to be a useful marker for prognosis in patients with chest pain symptoms and coronary artery calcification.

## Data availability statement

The raw data supporting the conclusions of this article will be made available by the authors, without undue reservation.

## Author contributions

X-ML and Y-NY: funding and conception and design of the study. Y-JQ and J-YL: literature search, data collection, and analysis of the manuscript. FL, X-XT, LZ, and Z-RZ: literature search, data collection, and processing. All authors read and approved the final manuscript.

## Conflict of interest

The authors declare that the research was conducted in the absence of any commercial or financial relationships that could be construed as a potential conflict of interest.

## Publisher’s note

All claims expressed in this article are solely those of the authors and do not necessarily represent those of their affiliated organizations, or those of the publisher, the editors and the reviewers. Any product that may be evaluated in this article, or claim that may be made by its manufacturer, is not guaranteed or endorsed by the publisher.
